# Sepsis induced changes of adipokines and cytokines - septic patients compared to morbidly obese patients

**DOI:** 10.1186/1471-2482-10-26

**Published:** 2010-09-09

**Authors:** Andreas Hillenbrand, Uwe Knippschild, Manfred Weiss, Hubert Schrezenmeier, Doris Henne-Bruns, Markus Huber-Lang, Anna M Wolf

**Affiliations:** 1 Department of General-, Visceral-, and Transplantation Surgery, University Hospital of Ulm, Steinhoevelstr. 9, 89075 Ulm, Germany; 2 Department of Anesthesiology University Hospital of Ulm, Steinhoevelstr. 9, 89075 Ulm, Germany; 3 Institute of Clinical Tranfusion Medicine and Immunogenetics, Helmholtzstr. 10; D-89081 Ulm; Germany; 4 Department of Traumatology, Hand and Reconstructive Surgery, University Hospital of Ulm, Steinhoevelstr. 9, 89075 Ulm, Germany

## Abstract

**Background:**

Hyperglycemia and insulin resistance frequently occur in critically ill and in morbidly obese (MO) patients. Both conditions are associated with altered serum levels of cytokines and adipokines. In addition, obesity related alterations in adipokine expression contribute to insulin resistance in metabolic syndrome. In this study we examined the serum adipocytokine profile in critically ill patients, MO patients, and healthy blood donors.

**Methods:**

33 patients who fulfilled the clinical criteria for severe sepsis or septic shock (SP) were prospectively enrolled in this study. A multiplex analysis was performed to evaluate plasma levels of adiponectin, resistin, leptin, active PAI-1, MCP-1, IL-1 alpha, IL-6, IL-8, IL-10, and TNF-alpha in 33 critically ill patients, 37 MO patients and 60 healthy blood donors (BD).

**Results:**

In SP, adiponectin was significantly lowered and resistin, active PAI-1, MCP-1, IL-1 alpha, IL-6, IL-8, IL-10, and TNF-alpha were significantly elevated compared to BD. Leptin levels were unchanged. In MO, adiponectin and IL-8 were significantly lowered, leptin, active PAI-1, MCP-1, IL-1 alpha, IL-6, and IL-10 significantly elevated, whereas resistin was unaltered.

In SP, adiponectin correlated negatively with BMI, SAPS II and SOFA scores, while resistin correlated positively with SAPS II and SOFA scores and leptin correlated positively with the BMI. Adiponectin was approximately equally diminished in SP and MO compared to BD. With the exception of active PAI-1, cytokine levels in SP were clearly higher compared to MO.

**Conclusion:**

A comparable adipocytokine profile was determined in critically ill and MO patients. As in MO, SP showed reduced adiponectin levels and elevated MCP-1, active PAI-1, IL-1 alpha, IL-6, and IL-10 levels. Leptin is only elevated in MO, while resistin, IL-8, and TNF-alpha is only elevated in SP. As in MO patients, increased levels of proinflammatory cytokines and altered levels of adipokines may contribute to the development of insulin resistance in critically ill patients.

## Background

Sepsis is defined as a systemic inflammatory response syndrome to infection, which, when associated with one or more organ system dysfunctions, is considered as severe sepsis. When sepsis is associated with shock, which is refractory to fluid resuscitation, the patient is considered to be in septic shock [1]. This is associated with an increased production of both pro- and anti-inflammatory cytokines [2]. Cytokines are low-molecular-weight polypeptides or glycoproteins that play an important role in regulating host response to infection, immune responses, inflammation, and trauma.

Some cytokines clearly promote inflammation and are called *proinflammatory cytokines*, whereas other cytokines suppress the activity of proinflammatory cytokines and are called *anti-inflammatory cytokines*.

Tumor necrosis factor (TNF) and interleukin (IL)-1α are proinflammatory cytokines, which can cause fever, inflammation, tissue destruction, and, in some cases, shock and death [3]. IL-10, a potent anti-inflammatory cytokine [4], is a key regulator of the immune system and is essential for homeostasis of the immune system [5]. IL-6, secreted by T cells and macrophages to stimulate immune response, is an interleukin that can exhibit both, pro-inflammatory and anti-inflammatory functions.

Another class of proinflammatory peptides are chemokines that selectively recruit monocytes, neutrophils, and lymphocytes and facilitate the passage from the circulation into the tissues. The prototypical chemokine is the neutrophil chemoattractant IL-8 [6]. Monocyte chemoattractant protein-1 (MCP-1) is another key chemokine that regulates migration and infiltration of monocytes and macrophages [7].

Many of the above mentioned cytokines such as IL-6 or IL-8 are not only synthesized by blood cells, but also by adipose tissue [8]. Particularly IL-6 could provide a potential link between fatty tissue and systemic inflammation [9].

In contrast with classical views, adipose tissue not only provides a depot for fat storage but has been increasingly recognized as an important endocrine organ, manufacturing adipokines, bioactive molecules including adiponectin, leptin, and resistin, and proinflammatory cytokines [10]. Below, the adipose tissue derived hormones adiponectin, leptin, and resistin are termed adipokines.

Whereas adiponectin and resistin are involved in glucose metabolism and exhibit anti-inflammatory features, leptin plays an important role in feeding behavior and exhibits proinflammatory features [11]. Plasminogen activator inhibitor-1 (PAI-1), secreted by adipose tissue has been shown to be an important inhibitor of fibrinolysis.

The expressions of these adipokines and cytokines are changed in obese persons, since obesity is associated with the appearance of a chronic, low inflammatory state due to changes in adipocyte and macrophage function [12]. Obesity leads to lowered levels of the anti-inflammatory adipokine adiponectin in circulation [13]. Adiponectin is known to be a regulator of insulin sensitivity and glucose metabolism. Furthermore, hypoadiponectinemia has been shown to be associated with insulin resistance and hyperinsulinemia [14]. Insulin resistance is one key feature of "metabolic syndrome", characterized by a distinct combination of metabolic disorders such as elevated triglycerides, reduced high densitiy lipoproteins, and/or enhanced fasting plasma glucose levels [15]. As obesity and metabolic syndrome, severe sepsis and septic shock are linked to increased blood glucose levels and insulin resistance even without a previous history of diabetes [16].

Several studies have provided evidence that increased blood glucose levels impair morbidity and mortality in critically ill patients and that intensive insulin therapy aimed at maintaining euglycemia markedly improved the outcome of these patients [17].

The aetiology of the "insulin resistance syndrome" caused by sepsis or metabolic syndrome is multifactorial, and the mechanisms underlying the intercorrelations among these metabolic conditions are not entirely clear. Although the involvement of adipose tissue hormones in the obesity-induced insulin resistance has been studied, there is only few information about its changes in critically ill patients [18].

Therefore, the present study was performed to determine a serum adipocytokine profile in critically ill patients which was subsequently compared with the profile of morbidly obese patients and a healthy control group. Further, we investigated if concentrations of adipocytokines are shifted in the same direction in critically ill septic patients as in morbidly obese patients compared to healthy controls. If so, which group has higher resultant changes? These results might increase our knowledge whether underlying mechanisms for insulin resistance are comparable in both groups of patients.

## Methods

### Patients and controls

#### Septic patients

33 patients in intensive care who fulfilled the clinical criteria for severe sepsis or septic shock were prospectively enrolled in this study. The criteria for severe sepsis and septic shock were in accordance with those defined by Bone [19]. The Simplified Acute Physiology Score (SAPS II) and Sequential Organ Failure Assessment score (SOFA) without Glasgow coma scale (GCS) were used to define the severity of disease and organ dysfunctions, respectively [20-22]. The underlying causes of sepsis were intestinal perforation (e.g. perforated ulcer, perforated diverticular disease; n = 11), inflammation (e.g. necrotizing pancreatitis, necrotizing fasciitis; n = 11), postoperative complication (e.g. infections, fistula, anastomosis-insufficiency, n = 7), ischemia (n = 2), and trauma (n = 2). The 33 septic patients (SP) had median (range) SAPS II and SOFA scores of 40 (17-60) and 9 (3-14), respectively. Seven patients had pre-existing type II diabetes.

Blood samples were collected one day after diagnosis of severe sepsis or septic shock at 5 a.m. in the fasting state (10 ml venous blood, collected in a chilled syringe with EDTA).

Serum was obtained by centrifugation (1800×*g *for 15 min), and the samples were subsequently stored in aliquots at -80°C until further analysis.

Body weight and height for calculation of body mass index (BMI) of septic patients was self reported (or estimated if no self-report was possible).

### Morbidly obese patients and controls

37 consecutive patients operated for morbid obesity (MO) at the author's hospital and 60 healthy blood donors (BD) were enrolled as the group of morbidly obese patients and as the control group, respectively. Blood samples from patients diagnosed with MO were collected preoperatively at 7 a.m. in fasting state. Samples of BD were taken at various times in no fasting state (10 ml venous blood, collected in a chilled syringe with EDTA). Anthropometric data (age and BMI) of patients of all three groups are listed in table 1.

**Table 1:** Gender specific anthropometric data (age and BMI) of patients with severe sepsis or septic shock (SP), morbidly obesity (MO), and healthy control group (BD).

	Age [years] (median; range)	BMI [kg/m^2^] (median; range)
**SP (n = 33)**	66 years (30 - 87)	26 kg/m^2 ^(18 - 37)

Female patients (n = 12)	81 years (42 - 87)	26 kg/m^2 ^(20 - 32)
Male patients (n = 21)	65 years (31 - 85)	26 kg/m^2 ^(18 - 37)

**MO (n = 37)**	45 years (17 - 59)	52 kg/m^2 ^(33 - 78)

Female patients (n = 24)	46 years (23 - 59)	52 kg/m^2 ^(35 - 78)
	
Male patients (n = 13)	42 years (17 - 59)	49 kg/m^2 ^(33 - 70)

**Control group (n = 60)**	45 years (19 - 71)	Not ascertained

Female control (n = 30)	44 years (19 - 60)	Not ascertained
Male control (n = 30)	46 years (19 - 71)	Not ascertained

The study was performed with the permission of the independent local ethics committee of the University of Ulm (approvals 112/2003 and 73/2009). An informed consent of all patients was necessary. If the patient was not capable of making decisions because of sedation or altered mental state, the informed consent was obtained after recovery. Exclusion criteria were: age < 18 years, pregnancy, rapidly progressing underlying disease, HIV/AIDS, cardiogenic shock as the primary underlying disease, underlying hematologic disease, or cytotoxic therapy given within the previous 6 months.

### Cytokine and adipocytes-derived hormones measurement and reagents

Multiplex analysis kits for IL-1α, IL-6, IL-8, IL-10, TNFα, MCP-1, active PAI-1, leptin, resistin, adiponectin were obtained from Millipore, Hamburg, Germany. In brief, the appropriate cytokine standards and samples, diluted in plasma dilution buffer, were added to wells of a filtered plate. The samples were incubated with 50 μl of the antibody -coupled microsphere set on a plate shaker in the dark at room temperature for 30 min. The samples were washed three times with 100 μl wash puffer. Freshly diluted secondary detection antibody (25 μl/well) was added to the wells and then incubated on a plate shaker in the dark at room temperature. Thereafter, samples were washed three times with 100 μl wash buffer. Fifty microliters of strepavidin-PE (16 μg/ml in assay buffer) was added to each well, and incubation continued on a plate shaker at room temperature for the first 10 min. Unbound analytes were filtered through the wells using the vacuum manifold. The bound beads were washed three times with 100 μl wash buffer. After the last wash step, 125 μl of assay buffer was added to each well and the plate was placed on a plate shaker set at 500 rpm for 1 min and then for 3 min at a reduced speed of 300 rpm. Finally, samples were analyzed on the Luminex system in accordance with the manufactures' instruction. As far as reaching the lowest traceable values, the sensitivity of the method was 0.3 ng/ml for leptin and 9.2 pg/ml for IL-1α.

### Statistical analysis

All values were expressed as median with range.

When comparing data between the three groups, i. e., SP, MO and BD, statistical analysis was performed using the Mann-Whitney U test. Analysis was performed with WinSTAT, Version 2009.1 (R. FitchSoftware).

The Spearman rank-order correlation coefficient was calculated for correlation analysis. R indicates the correlation coefficient. A value of p < 0.05 was considered statistically significant. Correlation coefficient values between 0.3 and 0.7 (-0.3 and -0.7) indicate a moderate, values between 0.7 and 1.0 (-0.7 and -1.0) indicate a strong positive (negative) linear relationship. No adjustment for multiple testing was done.

## Results

Adipocytokine levels are clearly changed in septic and obese patients. For a better overview, serum concentrations of adiponectin, leptin, resistin, TNF-α, MCP-1, active PAI-1, IL-1α, IL-6, IL-8, IL-10 are shown in table 2 and figs. 1, 2, 3, 4, 5, 6, 7, 8, 9 and 10.

**Table 2:** Gender specific adiponectin and leptin levels and resistin, MCP-1, active PAI-1, TNF-α, IL-1α, IL-6, IL-8, and IL-10 levels of septic patients (SP), morbidly obese (MO) and controls (BD).

Parameters (normal values)	SP (n = 33) (Median; range)	MO (n = 37) (Median; range)	BD (n = 60) (Median; range)	Change *SP vs. BD*	Change *MO vs. BD*
**Adiponectin** (μg/ml)	**10.3** (2.9 - 47.6)	**9.4** (3.8 - 57.8)	**14.7** (5.7 - 54.0)	**↓** p = < 0.01	**↓** p = < 0.01

Adiponectin male (μg/ml)	**9.5** (2.9 - 32.6)	**8.3** (3.8 - 12.3)	**11.8** (5.7 - 30.7)	**↓** p = 0.04	**↓** p = < 0.01

Adiponectin female (μg/ml)	**13.2** (5.3 - 47.6)	**12.4** (5.3 - 57.8)	**22.5** (9.8 - 54.0)	**↓** p = 0.02	**↓** p = < 0.01

**Leptin **(ng/ml)	**6.3** (0.3 - 80.7)	**45.0** (1.7 - 97.5)	**10.8** (0.3 - 58.5)	no significant change	**↑** p = < 0.01

Leptin male (ng/ml) (< 7 ng/ml)	**6.3** (0.3 - 24.7)	**34.2** (1.7 - 90.9)	**4.6** (0.3 - 19.1)	no significant change	**↑** p = < 0.01

Leptin female (ng/ml) (< 18 ng/ml)	**8.2** (0.9 - 80.7)	**47.3** (16.1 - 97.5)	**15.8** (0.7 - 58.5)	no significant change	**↑** p = < 0.01

**Resistin (ng/ml)**	**78.1** (13.4 - 52.3)	**9.9** (3.7 - 83.6)	**10.8** (5.2 - 24.9)	**↑** p = < 0.01	no significant change

**MCP-1 (pg/ml)**	**381.5** (72.3 - 887.3)	**108** (39.6 - 293)	**43.9** (12.1 - 150)	**↑** p = < 0.01	**↑** p = < 0.01

**Active PAI-1 (ng/ml)**	**35.9** (11.5 - 160.3)	**57.5** (3.7 - 176.7)	**6.0** (2.4 - 31.3)	**↑** p = < 0.01	**↑** p = < 0.01

**TNF-α (pg/ml)**	**13.1** (2.4 - 65.5)	**4.0** (2.4 - 26.8)	**3.9** (1.2 - 10.9)	**↑** p = < 0.01	no significant change

**IL-1α (pg/ml)**	**68.6** (9.2 - 203.3)	**59.4** (9.2 - 210.0)	**11.0** (9.2 - 168)	**↑** p = 0.04	**↑** p = 0.01

**IL-6 (pg/ml)**	**163.6** (14.4 - 10000)	**2.9** (0.9 - 64.2)	**0.7** (0.4 - 568.6)	**↑** p = < 0.01	**↑** p = < 0.01

**IL-8 (pg/ml)**	**59.4** (4.6 - 1335.6)	**3.7** (0.3 - 38.8)	**9.8** (0.9 - 126.7)	**↑** p = < 0.01	**↓** p = < 0.01

**IL-10 (pg/ml)**	**26.7** (3.9 - 276.7)	**2.5** (2.4 - 8.6)	**1.2** (0.9 - 1038)	**↑** p = < 0.01	**↑** p = 0.01

**↓↑: **Significant increase/decrease;

**Figure 1:**
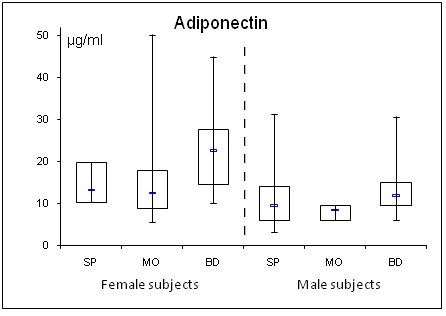
**Gender specific serum concentrations of adiponectin in septic (SP) and morbidly obese (MO) patients and healthy controls (BD)**. The top and bottom of the rectangle represent the 25th and 75th percentile. The line within the rectangle represents the median. The whiskers extend from the 5th percentile to the 95th percentile.

**Figure 2:**
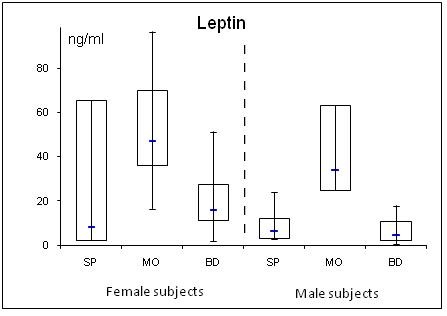
**Gender specific serum concentrations of leptin in septic (SP) and morbidly obese (MO) patients and healthy controls (BD)**.

**Figure 3:**
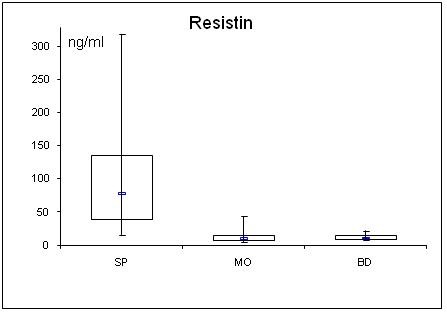
**Serum concentrations of resistin in septic (SP) and morbidly obese (MO) patients and healthy controls (BD)**.

**Figure 4:**
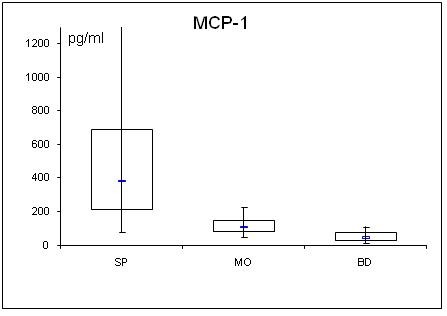
**Serum concentrations of MCP-1 in septic (SP) and morbidly obese (MO) patients and healthy controls (BD)**.

**Figure 5:**
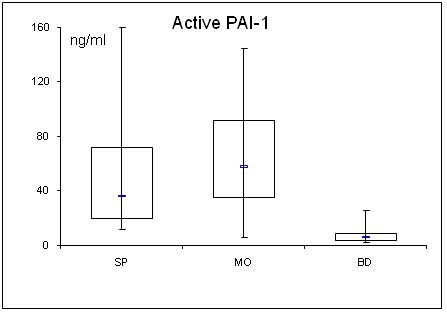
**Serum concentrations of active PAI-1 in septic (SP) and morbidly obese (MO) patients and healthy controls (BD)**.

**Figure 6:**
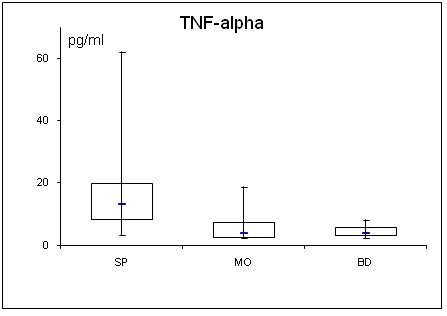
**Serum concentrations of TNF-α in septic (SP) and morbidly obese (MO) patients and healthy controls (BD)**.

**Figure 7:**
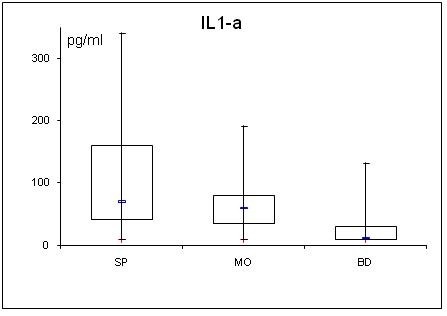
**Serum concentrations of IL-1a in septic (SP) and morbidly obese (MO) patients and healthy controls (BD)**.

**Figure 8:**
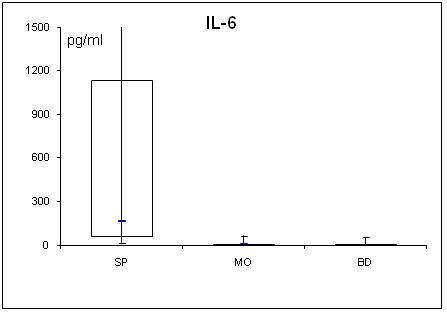
**Serum concentrations of IL-6 in septic (SP) and morbidly obese (MO) patients and healthy controls (BD)**.

**Figure 9:**
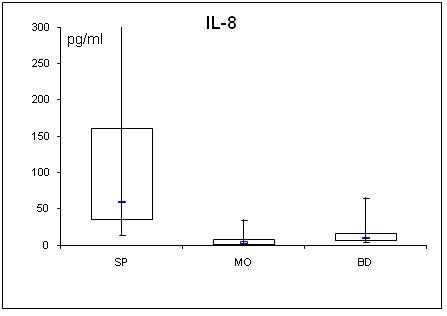
**Serum concentrations of IL-8 in septic (SP) and morbidly obese (MO) patients and healthy controls (BD)**.

**Figure 10:**
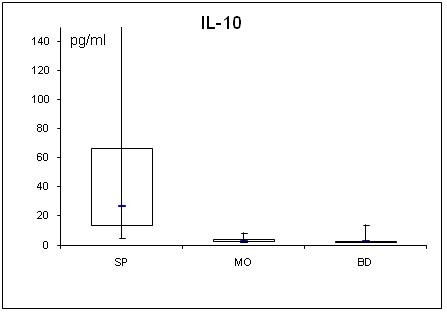
**Serum concentrations of IL-10 in septic (SP) and morbidly obese (MO) patients and healthy controls (BD)**.

### Adipokines (Adiponectin, Resistin, and Leptin)

In SP, adiponectin was lowered and resistin elevated compared to BD. Further, adiponectin correlated negatively with the SAPS II (r = -0.30; p = 0.043) and SOFA scores (r = -0.31; p = 0.042), while resistin correlated positively (SAPS II: r = 0.35; p = 0.023; SOFA: r = 0.30; p = 0.045). Leptin levels were unchanged in SP compared to BD. BMI correlated negatively with adiponectin (r = 0.32; p = 0.036) and positively with the leptin levels (r = 0.64; p = < 0.001) in SP. In MO, gender specific adiponectin was lowered and leptin elevated compared to BD. Resistin was unaltered. Adiponectin was equally diminished in SP and MO compared to BD.

As in BD and MO, there was a profound gender difference in adiponectin levels between male and female SP (male median levels: 9.3 μg/ml vs. female median levels: 13.2 μg/ml; p = 0.017).

### Cytokines (TNFα, MCP-1, active PAI-1 and interleukins)

All determined cytokines had significantly higher values in SP compared to BD. In MO, IL-8 was the only assessed value that showed a significant fall compared to BD, all other cytokines in MO were significantly elevated. With the exception of active PAI-1, cytokine levels in SP were clearly higher compared to MO.

Again, in SP there were positive correlations of active PAI-1, IL-6, IL-8, MCP-1, and IL-10 with SAPS II and SOFA scores. IL-1α and TNF-α showed no correlation with SAPS II and SOFA scores. P-values and correlation coefficients with SAPS II and SOFA scores are: active PAI-1 (p = 0.032, r = 0.33; p = 0.021. r = 0.34), IL-6 (p = 0.006, r = 0.45; p = 0.030, r = 0.39), IL-8 (p = 0.032, r = 0.31; p = 0.012, r = 0.41), MCP-1 (p = 0.082, r= 0.29; p = 0.043, r = 0.33), IL-10 (p = 0.040, r = 0.28; p = 0.074, r = 0.26), IL-1α (p = 0.118, r = -0.21; p = 0.389, r = -0.05) and TNF-α (p = 0.252, r = 0.13; p = 0.419, r = 0.04).

### Age dependency of values

Since SP were significantly older compared to MO and BD patients, it is necessary to consider an age dependency of values.

In BD, female adiponectin showed certain correlation with age (r = 0.34; p = 0.032; male patients: r = -0.26; p = 0.085). There was no correlation with patients' age in BD concerning female/male leptin, resistin, active PAI-1, MCP-1, IL-1α, IL-6, IL-8; IL-10, and TNF-α. Values of MO and SP showed no correlation with age.

Seven patients of the sepsis group had preexisting type 2 diabetes. These seven patients showed no difference in adiponectin, resistin, leptin, active PAI-1, MCP-1, IL-1α, IL-6, IL-8, IL-10, and TNF-alpha levels.

## Discussion

In this study we determined serum levels of classical cytokines and adipokines in septic and morbidly obese patients and compared them to healthy blood donors. The most important finding of this study is that both, SP and MO have similarly shifted values compared to a control group of healthy BD. Moreover, most cytokine concentrations were greater in sepsis patients than in the morbidly obese patients.

### Adipokines

Our results revealed that the only known adipose tissue derived factor with major insulin-sensitizing and anti-inflammatory properties, adiponectin, is equally diminished in SP and MO. Adiponectin is the most abundant protein produced by adipose tissue and its blood concentration is much higher than the concentration of other known hormones. Normal serum adiponectin levels are higher in women compared to men. Adiponectin attenuates inflammatory actions on several levels. It suppresses function of mature macrophages and inhibits growth of macrophage precursors [23]. Further, adiponectin attenuates the production of TNF-α and IL-6 production in macrophages and induces that of IL-10 [24]. Thus, the lower levels of adiponectin in the present study in the septic and morbid obese patients in comparison to healthy controls might have contributed to the higher levels of IL-6 and TNF-α. Otherwise, adiponectin has an insulin sensitizing effect. Adiponectin levels decrease in parallel with the progression of insulin resistance and type 2 diabetes [25], and the antidiabetic drug thiazolidinedione increases adiponectin levels [26]. Given the anti-inflammatory effects of adiponectin, it is plausible that lowered adiponectin levels may predispose to sepsis-related proinflammatory complications in states of obesity, diabetes, and insulin resistance. On the other hand, the reduced adiponectin levels in our sepsis patients might support insulin resistance. Several other studies also showed lower adiponektin levels in septic patients [27,28].

Besides adiponectin, resistin has been reported to participate in the inflammatory response [29]. Resistin was originally discovered in mice as an adipocytes derived hormone. It was found to be increased in obesity and causing insulin resistance in mice [30]. In contrast to mice, resistin in humans is mainly derived from macrophages rather than adipocytes [31]. In humans, resistin is a protein with proinflammatory properties. Its secretion is stimulated by inflammatory processes, glucocorticoids and lipopolysaccharides (LPS), whereas TNF-α and β-adrenergic stimulation act as inhibitors [32]. Resistin has been shown to increase transcriptional events leading to an increased expression of several pro-inflammatory cytokines including IL-1, IL-6, IL-12, and TNF-α [33]. In a positive feedback loop, resistin can be up-regulated by interleukins, and also by microbial antigens such as LPS [34]. In accordance with these reports, we found significantly higher resistin levels in SP, but not in MO, compared to healthy BD supporting the hypothesis that in humans resistin is predominantly a component of the systemic inflammatory response to infection.

Since pro-inflammatory cytokines were higher in SP than in MO, this could have led to the higher resistin levels in SP than in MO in our study. Otherwise, the higher resistin levels in SP compared to MO may have contributed to the higher pro-inflammatory cytokines in SP compared to MO. In our SP and MO patients, as well as in prior studies of septic patients [35,36], resistin did not correlate to obesity measured by BMI which suggests that in circumstances of critical illness the release of resistin by macrophages plays a superior role compared with the secretion from adipocytes. This could possibly also explain why there was no difference in resistin levels of patients with preexisting diabetes mellitus.

Leptin was the only measured adipokine in the present study that was marginally reduced in SP compared to BD. The MO group had significantly elevated levels. In the present study, normal leptin serum levels were higher in women compared to men. In the obese, increased fat storage will lead to enhanced leptin levels [37]. Leptin can affect glucose metabolism and increases insulin sensitivity. Obese humans are often insulin- and leptin-resistant [38]. The role of leptin in sepsis and septic shock is controversial. Earlier reports suggested that high lepin levels are associated with increased survival in sepsis and septic shock [39,40], several other reports -such as our study- fail to show a correlation between leptin and sepsis [41]. Our minor changes of leptin serum levels in SP are in line with a reported temporal decrease in leptin after operative stress followed by a subsequent rise slightly above the initial levels [42].

### Cytokines

Several studies suggest that TNF-α and IL-6 are both involved in obesity-related insulin resistance and that TNF-α is one of the most important mediators of inflammation [43]. TNF-α is not secreted by adipocytes but by infiltrating macrophages in adipose tissue, whereas adipose tissue is a significant source of IL-6 [44]. Expression and secretion of TNF-α increases with obesity and correlates positively with body mass index [45]. TNF-α and IL-6 are known to promote lipolysis and the secretion of free fatty acids, which contributes to an increase in hepatic glucose production and insulin resistance [46]. On a cellular level, TNF-α is a potent inhibitor of the insulin-stimulated tyrosine phosphorylations on the beta-chain of the insulin receptor and insulin receptor substrate-1, suggesting that TNF-α may play a crucial role in the systemic insulin resistance of non insulin dependent diabetes mellitus [47]. In analogy, the elevated IL-6 levels in our septic and morbidly obese patients may contribute to insulin resistance. In contrast to the morbidly obese patients, in addition, the elevated TNF-α levels in our septic patients might play a role in insulin resistance.

Human fat cells are also known to produce proinflammatory IL-8 which was increased in insulin-resistant subjects [48]. Surprisingly, we found reduced levels of IL-8 in MO compared to BD. Serum levels of MO patients (median: 3.7 pg/ml) match reported levels in the obese. Serum level of BD seems (median: 9.8 pg/ml) markedly elevated compared to reported levels of approximately 3.2 pg/ml [49]. Insofar, reported IL-8 levels should be interpreted with care. Nevertheless, the higher IL-8 levels in septic patients compared to the morbidly obese patients underline the infection induced origin of IL-8 in our study.

The presented study has several limitations. In the healthy control group, there was no investigation of hidden or not yet clinically manifest side diagnoses. Consequently, there is no information about type 2 diabetes or other chronic inflammatory diseases without clinical manifestation. Moreover, the control group was significantly younger than the sepsis group and blood samples were not taken in fasting state in the control group. However, only leptin showed a significant correlation with the blood donor's age in the control group, whereas resistin, IL-1α, IL-6, and IL-8 did not. Thus, the differences between these values in the septic patients group and control group may be less striking. Further, no weight/height for BMI calculation was ascertained in BD group. Another crucial points are the small number and the heterogeneousness of the septic group including patients with different cause of sepsis. However, this reflects the typical clinical situation and case mix in intensive care units.

It is also important to note that many other factors in addition to proinflammatory cytokines such as cortisol, catecholamines, growth hormone, and other stress-related factors can significantly contribute to the development of insulin resistance in critically ill patients [50].

## Conclusion

In conclusion, we could determine a sepsis specific adipocytokine profile in critically ill patients. In comparison with levels in a healthy control group, we observed a marked reduction of adiponectin and a profound increase of resistin, active PAI-1, MCP-1, IL-1α, IL-6, IL-8, IL-10, and TNF-α in septic patients. As in SP, MO showed reduced adiponectin levels and elevated MCP-1, active PAI-1, IL-1α, IL-6, and IL-10 levels. Leptin is only elevated in MO, while resistin, IL-8, and TNF-α is only elevated in SP. Taken together, the finding of increased levels of proinflammatory cytokines and altered levels of adipokines underlines its possible contribution in the development of insulin resistance in critically ill patients with sepsis.

## Competing interests

The authors declare that they have no competing interests.

## Authors' contributions

All authors have contributed substantially to the submitted work and have read and revised the manuscript and approved the final version. In particular AH participated in the design of the study, data acquisition, analysis and drafting of the manuscript. UK participated in data acquisition, analysis and drafting of the manuscript. MW assessed and recorded sepsis scores and participated in the drafting of the manuscript, HS took blood samples of control group, DHB initialized the work, provided laboratory results and gave approval for submission. AMW and MHL provided general idea, initialized the work, took blood samples, provided laboratory results and got *ethical approval*.

## Pre-publication history

The pre-publication history for this paper can be accessed here:

http://www.biomedcentral.com/1471-2482/10/26/prepub
